# The clinical features of radiation cataract in patients with ocular adnexal mucosa-associated lymphoid tissue lymphoma

**DOI:** 10.1186/s13014-018-1045-7

**Published:** 2018-05-16

**Authors:** Kanae Fukutsu, Satoru Kase, Kan Ishijima, Rumiko Kinoshita, Susumu Ishida

**Affiliations:** 1 Department of Ophthalmology, Hokkaido University Graduate School of Medicine, N-15, W-7, Kita-ku, Sapporo, 060-8638 Japan; 2 Department of Radiation Oncology, Hokkaido University Hospital, Sapporo, Japan

**Keywords:** Ocular adnexa, MALT lymphoma, Female, Bolus, Radiation cataract

## Abstract

**Background:**

To examine the clinical features of radiation cataract in patients with ocular adnexal mucosa-associated lymphoid tissue (MALT) lymphoma.

**Methods:**

Twenty-one patients with 26 eyes diagnosed with ocular adnexal MALT lymphoma (26 eyes), who were treated in Hokkaido University Hospital, were retrospectively reviewed based on medical records.

**Results:**

Out of the 21 patients, 16 patients (21 eyes) received radiation therapy (RT) with a total dose of 30 Gy. All cases eventually achieved complete remission. Eight of these patients (11 eyes: 52.3%) required cataract surgery after RT. The mean age at surgery was 56.8 (40–70) years. The mean latency between RT and the indication for surgery was 43.3 months. The percentage of females was significantly higher in patients who required surgery (*P* < 0.01), compared with those without surgery. The eyes of patients who received bolus technique on radiation treatment developed cataract more frequently (*P* < 0.05). In contrast, none of the patients without RT required cataract surgery.

**Conclusions:**

Patients with ocular adnexal MALT lymphoma who underwent surgery for radiation cataract were seen more often in relatively young, female patients, and surgery was required about 3 years after RT. A long-term observation may be needed for patients after RT for a tumor. A female sex and the bolus technique may be risk factors for radiation cataract.

## Background

Mucosa-associated lymphoid tissue (MALT) lymphoma is the most common subtype among primary ocular adnexal lymphomas, with a typically indolent nature [1]. Several reports have shown the efficacy of radiation therapy (RT) against ocular adnexal MALT lymphoma, offering excellent local control with a favorable clinical course [2–4]. Indeed, the 5-year survival rate is known to be as high as 90–100% [2–4]. However, besides the efficacy, side effects such as cataract, dry eye, and retinopathy occur following radiotherapy [5].

Lens shielding and limitation of the radiation dose help reduce the risk of cataract formation [6–8]. However, the details of radiation cataract including the latency of cataract formation, type of cataract, characteristics of patients, and correlation with radiotherapy technique remain largely unknown in patients with ocular adnexal MALT lymphoma. The aim of this study was to examine the clinical features of radiation cataract in patients with MALT lymphoma.

## Methods

### Patients

This was a retrospective observational case study. The Institutional Review Board of Hokkaido University Hospital for clinical research approved this study (IRB number: 015–534). In this study, 21 patients (26 eyes) with ocular adnexal MALT lymphoma were reviewed, who were treated in Hokkaido University Hospital, Department of Ophthalmology, Sapporo, Japan, from Nov. 2004 to Dec. 2011. We also found another patient who indicated the development of cataract during the period; however, the patient was excluded in this study because of a severe dementia, and there was a difficulty to obtain reliable data such as visual acuity before and after the radiotherapy. Of those 21 patients, 16 eventually elected to receive RT after being advised by the physicians of risks and benefits. Medical records for eye exams were reviewed for those 16 in the RT cohort to study. Based on medical records, we reviewed their clinical findings such as visual acuity, type of cataract and fundus findings after dilatation of pupils, and radiation dose. The diagnosis of MALT lymphoma was made based on the findings from slit-lamp examination, imaging modalities, pathological examination, and immunoglobulin heavy chain gene rearrangement. We determined the tumor stage based on Ann Arbor staging [6]. For imaging, we used CT or MRI to classify the lymphoma as follows: when a tumor was absent inside the orbit but present on the surface of the conjunctiva, it was classified as the conjunctival type. If a tumor was present inside the orbit, it was classified as the orbital type. The patients who received radiotherapy were divided into two groups: those who suffered from blurred vision and required cataract surgery were classified as a cataract group; those who did not show significant cataract development nor require surgery were classified as a non-cataract group.

### Radiotherapy

Radiotherapy was delivered using photon or electron beams. All photon-treatment planning were performed with 3-dimensional computed tomographic simulation using the XiO treatment planning system (ver. 4.1.1–4.6.0, Elekta, Stockholm, Sweden). Patents were immobilized using head shells. The electron field was defined manually by physician. The energy of the beam was selected to cover the tumor. A bolus was used to compensate for the surface dose as needed. RT techniques were carefully considered by physicians to ensure adequate dose delivering to the tumor while minimizing the dose to normal tissues such as the retina and macula. Treatment were delivered using EXL-20DP (Mitsubishi Electronics, CO.,Ltd., Tokyo, Japan) or MHCL-15SP (Mitsubishi Electonics Co., Ltd., Tokyo, Japan) or Clinac 2100 (Varian Medical Systems, Palo Alto, CA, USA) or Clinac CL-iX (Varian medical Systmes, Palo Alto, CA, USA) Linac.

### Statistical analysis

Between the cataract and non-cataract groups, we compared their mean age at diagnosis, population of each sex, and the type of lymphoma. We applied the Mann-Whitney U test to compare the mean ages. We used the Chi-square test to compare the populations of sexes, type of lymphoma and radiation, and use of bolus technique at radiation between the two groups. A *p*-value of < 0.05 was considered significant. We performed all statistical analyses using statistical analyses software, Ver. 2.0, for Macintosh (Statistics Survey System-development, Esumi Corporation, Tokyo, Japan: http://www.esumi.co.jp).

## Results

### Clinical features of MALT lymphoma and RT regimen

Six males and 15 females were eligible. MALT lymphomas with the conjunctival and orbital types were seen in 16 eyes of 14 patients, and 10 eyes of 7 patients, respectively. Radiotherapy was administered to 21 eyes of 16 patients. Most of them were diagnosed as stage IE or IIE, and only patient was diagnosed as stage III. All orbital types were treated by photon beam. The prescribed dose was 30.6 Gy in 17 fractions for one eye, and 30 Gy in 15 fractions for the other 20 eyes. Eleven eyes of 5 patients received 4 or 6MV photon beams. Of those 5 patients, 6 eyes of 3 patients with bilateral disease were treated with an opposed lateral beam arrangement. The remaining 2 unilateral patients were treated with an anterior beam and two oblique ports with wedge filters. The field size of photon treatment ranges from 4.6 cm collimator equivalent square to 6.8 cm. Ten eyes of 10 patients were treated with 4 or 6-MeV electron beams, forming a single anterior field. Electron treatment fields were shaped by insertion of lead to applicator. A bolus was used for 9 eyes of 7 patients to provide an adequate dose to the surface. Seven patients were instructed to keep the affected eye closed during irradiation (Table 1). The purpose of closing the eye was to increase radiation dose to the ocular surface, using patient’s eyelid as a bolus.

**Table 1:** Clinical profiles and regimen of radiation therapy (RT) in patients with mucosa-associated lymphoid tissue lymphoma

	Case	Eye	Sex	Age	Side	Type of MALT lymphoma	Type of RT	Energy	Total dose/fraction	Filed size	Eye	Use of bolus	Field arrangement
Cataract	1	1	F	63	R	conjunctival	electron	4-MeV	30Gy/15Fr	6cmW 5cmD	closed	(−)	single
	2	2	F	55	R	conjunctival	electron	6-MeV	30Gy/15Fr	7cmW 6cmD	not instructed	(+)	single
	3	3	F	55	L	conjunctival	photon	6-MV	30Gy/15Fr	6.0 cm Coll. Eq	not instructed	(+)	single
	4	4	M	53	R	orbital	photon	6-MV	30Gy/15Fr	6.9 cm,6.1 cm Coll.Eq	not instructed	(+)	Wedge pair
	5	5	F	45	R	conjunctival	photon	6-MV	30Gy/15Fr	4.6 cm、4.6 cm Coll.Eq	not instructed	(+)	opposing
		6	F	45	L	conjunctival	photon	6-MV	30Gy/15Fr	4.6 cm、4.6 cm Coll.Eq	not instructed	(+)	opposing
	6	7	F	65	R	orbital	photon	4-MV	30Gy/15Fr	5.0 cm、5.0 cm Coll.Eq	not instructed	(−)	opposing
		8	F	65	L	orbital	photon	4-MV	30Gy/15Fr	5.0 cm、5.0 cm Coll.Eq	not instructed	(−)	opposing
	7	9	F	36	R	conjunctival	electron	6-MeV	30Gy/15Fr	6cmW 5cmD	not instructed	(+)	single
		10	F	36	L	conjunctival	electron	6-MeV	30Gy/15Fr	6cmW 5cmD	not instructed	(+)	single
	8	11	F	63	R	conjunctival	electron	6-MeV	30.6Gy/17Fr	6cmW 5cmD	closed	(−)	single
Non-cataract	9	12	F	49	R	orbital	photon	6-MV	30Gy/15Fr	7.0 cm Coll. Eq	not instructed	(+)	opposing
		13	F	49	L	orbital	photon	6-MV	30Gy/15Fr	7.0 cm Coll. Eq	not instructed	(+)	opposing
	10	14	F	84	L	orbital	photon	6-MV	30Gy/15Fr	5.0 cm Coll. Eq	not instructed	(−)	single
	11	15	F	38	R	conjunctival	electron	4-MeV	30Gy/15Fr	5cmW 4cmD	closed	(−)	single
	12	16	F	32	R	conjunctival	electron	4-MeV	30Gy/15Fr	7cmW 6cmD	closed	(−)	single
	13	17	M	81	R	conjunctival	photon	4-MV	30Gy/15Fr	5.7 cm Coll. Eq	not instructed	(−)	opposing
		18	M	81	L	conjunctival	photon	4-MV	30Gy/15Fr	5.7 cm Coll. Eq	not instructed	(−)	opposing
	14	19	M	45	R	conjunctival	electron	4-MeV	30Gy/15Fr	7cmW 7cmD	closed	(−)	single
	15	20	M	50	R	conjunctival	electron	4-MeV	30Gy/15Fr	7cmW 6cmD	closed	(−)	single
	16	21	F	42	L	conjunctival	electron	4-MeV	30Gy/15Fr	5.5cmW 5.5cmD	closed	(−)	single

*M* male, *F* female, *R* right, *L* left, *Gy* Gray, *Coll*. *Eq* Collimator equevalent squere, *D* depth; single, single field irradiation; opposing, opposing portal irradiation

A lens shield was not applied for any patients. Table 1 shows the details of radiotherapy. The number of eyes that underwent photon and electron beam radiation was 6 and 5 in cataract group, and 5 and 5 in non-cataract group respectively. There were no significant deviations regarding photon vs. electron beam radiation related to cataractogenesis in this study (*P* > 0.05).

### Characteristics of cataract group

**Fig. 1:**
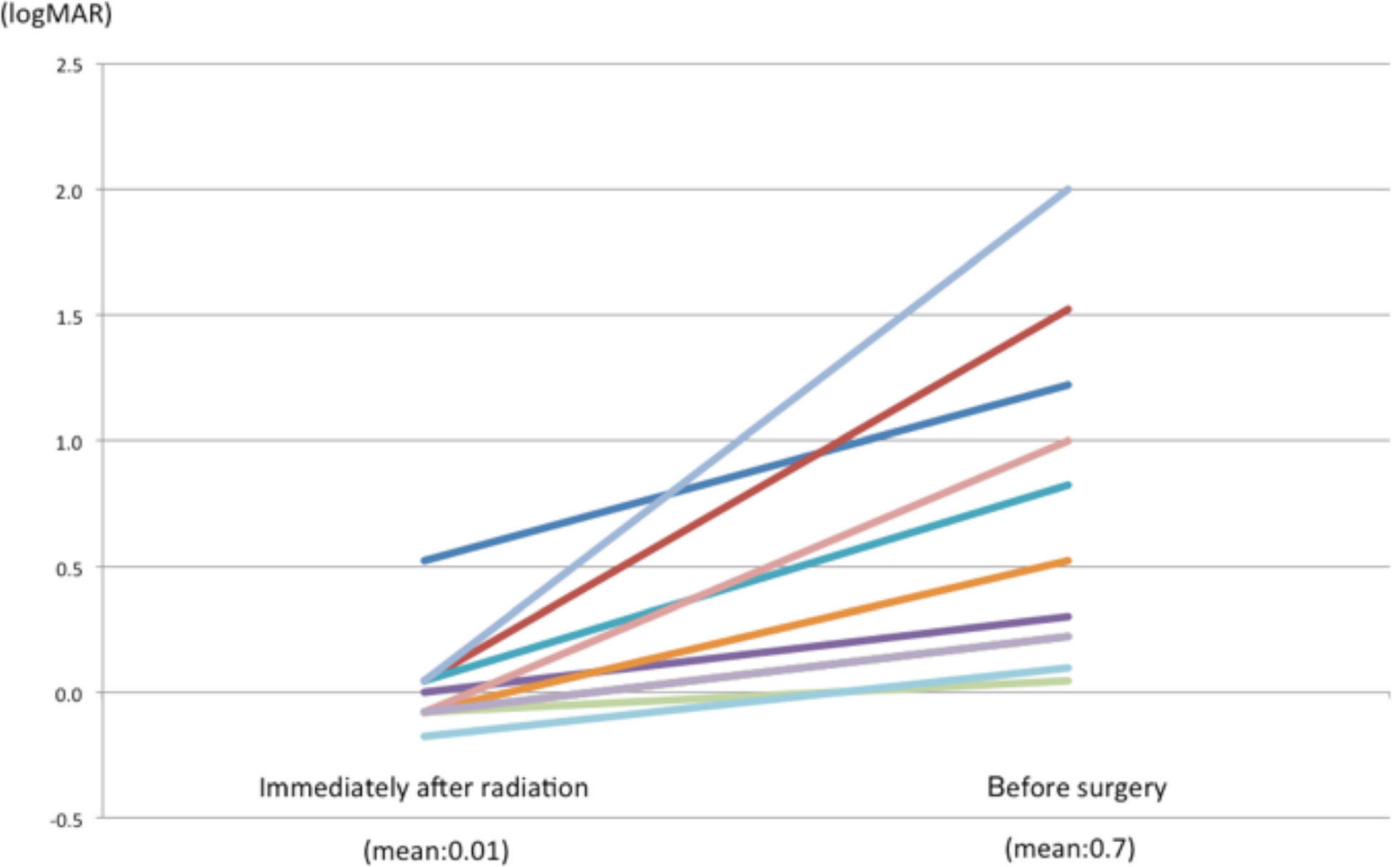
Changes of visual acuity in cataract group. The mean visual acuity in the cataract group immediately after the radiotherapy was 0.01 (logMAR). It dropped to 0.7 (logMAR) before the surgery

Among those who received radiotherapy, the median follow-up duration was 58.7 months. Eight patients (52.4%) (11 eyes) were classified as the cataract group (Table 1). Four eyes has had incipient cataract, one eye had a nuclear cataract (EL1), and the others had no cataract at the initiation of radiotherapy. In that group, mean age at the cataract surgery was 56.8 years. The mean latency between the final radiation and surgery was 43.3 months. We compared the visual acuity of the cataract group immediately after the radiotherapy and before the surgery. Although the mean logMAR visual acuity immediately after the radiotherapy was 0.01, it dropped to 0.7 before the surgery (Fig. 1). In the cataract group, posterior subcapsular cataract (PSC) was seen in 11 (100%) out of 11 eyes. The cataract extended to the lens nucleus was seen in 9 eyes. Among those who underwent cataract surgery, the zonule of Zinn was disrupted during surgery in one eye, resulting in out-of-the-bag fixation of intraocular lens. It was difficult to remove posterior capsular opacity in two eyes, which required laser capsulotomy after a couple of months. Other cases experienced no complications associated with the surgery.

### Comparison between cataract and non-cataract groups

In contrast, 8 patients (47.6%) (10 eyes) were classified as the non-cataract group. The age at the diagnosis of MALT lymphoma was 52.8 and 55.1 years in the cataract and non-cataract groups, respectively. One eye was from a male, and 10 eyes were from females in the cataract group, whereas 4 and 6 eyes were from males and females in the non-cataract group, respectively. The number of females was significantly higher in the cataract group than the non-cataract group (*P* < 0.01). The mean age of females was 52.8 and 53.4 years in the cataract and non-cataract groups respectively, with no significant difference between them. Eight eyes had conjunctival type MALT lymphoma and 3 eyes had the orbital-type in the cataract group, whereas 7 had the conjunctival type and 3 had the orbital type in the non-cataract group. There was no statistical deviation between conjunctival and orbital types regarding cataract/non-cataract group (*P* > 0.05), suggesting that the types of lymphoma might not affect the incidence of cataract formation. In this study, the bolus technique was used for seven eyes in the cataract group when they received the radiation treatment. On the other hand, two eyes of 10 eyes in the non-cataract group underwent this technique. The use of the bolus technique significantly affected the development of cataract (*P* < 0.05). Further, to eliminate the possibility of individual radiosensitivity, effect of the bolus technique on cataractogenesis was evaluated based on each patient. As shown in Table 1, eight patients were classified as cataract and non-cataract group, respectively. Among 8 patients in the cataract group, the bolus technique was used in 5 patients (62.5%), whereas the bolus technique was applied in 1 patient in the non-cataract group (12.5%). There was a statistically significant correlation between bolus technique application and the number of patients with cataract which required surgery (*P* < 0.05).

## Discussion

It is known that ocular toxicity can develop such as dry eye, cataract formation, retinopathy, and glaucoma following radiotherapy against ocular adnexal MALT lymphoma. Previous studies reported that approximately 55% of patients receiving radiotherapy without lens shielding showed significant cataract formation within 5–9 number of years following RT. [7, 8] In our study, lens shielding was not applied in any cases of radiotherapy, but cataract formation was noted in more than half of them (52.3%). These outcomes were consistent with findings reported previously [7, 8]. In another study, the mean age at cataract surgery was 46 years, and the mean latency from radiotherapy to surgery was 36.6 months [9]. Our results showed that the age was 56.8 years and the latency was 43.3 months. These findings indicate that radiation cataract in ocular adnexal MALT lymphoma can develop over a relatively long period, in relatively young patients. Moreover, this study analyzed the location of tumors and its correlation with cataract formation; however, the frequency of radiation cataract did not differ regardless of the type of lymphoma in this study.

It is likely, given the rather high dose of radiotherapy utilized, that all patients whose anterior lens was included in the radiation field, will eventually develop a radiation cataract [10, 11]. All the radiation cataract cases in ocular adnexal MALT lymphoma showed PSC according to a previous study [5]. Our data showed the similar results; all the eyes in the cataract group formed PSC. Moreover, the cataract extended to the lens nucleus was seen in 82% in this study. These results are consistent with the previous studies which have suggested that the radiation-induced cataracts begin in the posterior subcapsular location before spreading to the anterior subcapsular and nuclear locations [12–14]. The previous studies about radiation cataractogenesis suggest that a relatively low dose of irradiation led to lens epithelial cells dividing abnormally and migrating to the posterior pole of the lens, resulting in PSC formation [12, 13] and then gradually progress to the cortex and nucleus until they become indistinguishable from other types of cataracts [14].

It is known that vision-threatening radiation cataract formation in ocular adnexal MALT lymphoma can be reduced by using lens shielding, and/or limiting the dose to under 30.6 Gy [7, 8, 15, 16]. However, since inappropriate use of lens shielding leads to local failure, careful consideration is needed to use it [17]. None of our patients underwent lens shielding and the radiation dose was always limited to under 30.6Gy. Thus, considering the fact that all of our cases showed almost the same conditions, the significantly higher rate of females in the cataract group than in the non-cataract group could indicate that the sex may be one of the risk factors for time of onset in radiation cataract. Several population-based studies have shown that females have a higher prevalence of lens opacities [18]. Aina et al. analyzed 10,000 women, indicating that there is a relationship between estrogen, the hormone which decreases at menopause, and cataract formation in females [19]. The exact mechanism is unclear, but Ganatra et al. demonstrated that estrogen might protect the cytoskeleton of lens epithelial cells from hydrogen peroxide-induced oxidative stress [20]. There are some studies suggesting that there is a gender-related difference in radiation-induced cataractogenesis. One of them showed a high incidence of radiation cataract in male rats compared to female rats. However, that difference cannot be attributed to estrogen levels, although they expected that hormone levels had some effects on the cataractogenesis. The possibility of both negative and positive radioprotective effects of estrogen in irradiated rat eyes has been described. Overall, the exact mechanism how the sex hormone is affecting the radiation cataractogenesis remains controversial [7, 21, 22]. In our study, there was no significant difference between the mean ages of females in the cataract (52.8 years) and the non-cataract (53.4 years) groups, although those ages might be potentially correlated with post-menopausal. Further studies are needed to clarify the reason for the higher prevalence of females in the cataract group.

It is well known that the lens is very sensitive to radiation. Emami et al. estimated the TD5/5 (the probability of 5% complication within five years from treatment) of the lens to be 10Gy and the TD50/5 (the probability of 50% complication with five years from treatment) to be 18Gy [23]. Recent ICRP guidelines indicate the threshold of a radiation induced cataract is 0.5Gy [7]. The risk of cataract and the latent period between irradiation and the occurrence of cataract are dose dependent. Hall and Giaccia reported that the latent period and the risk of cataract progression were 8 years and 33% after 2.5 to 6.5Gy, and 4 years and 66% after 6.51 to 11.5Gy, respectively [24].

**Fig. 2:**
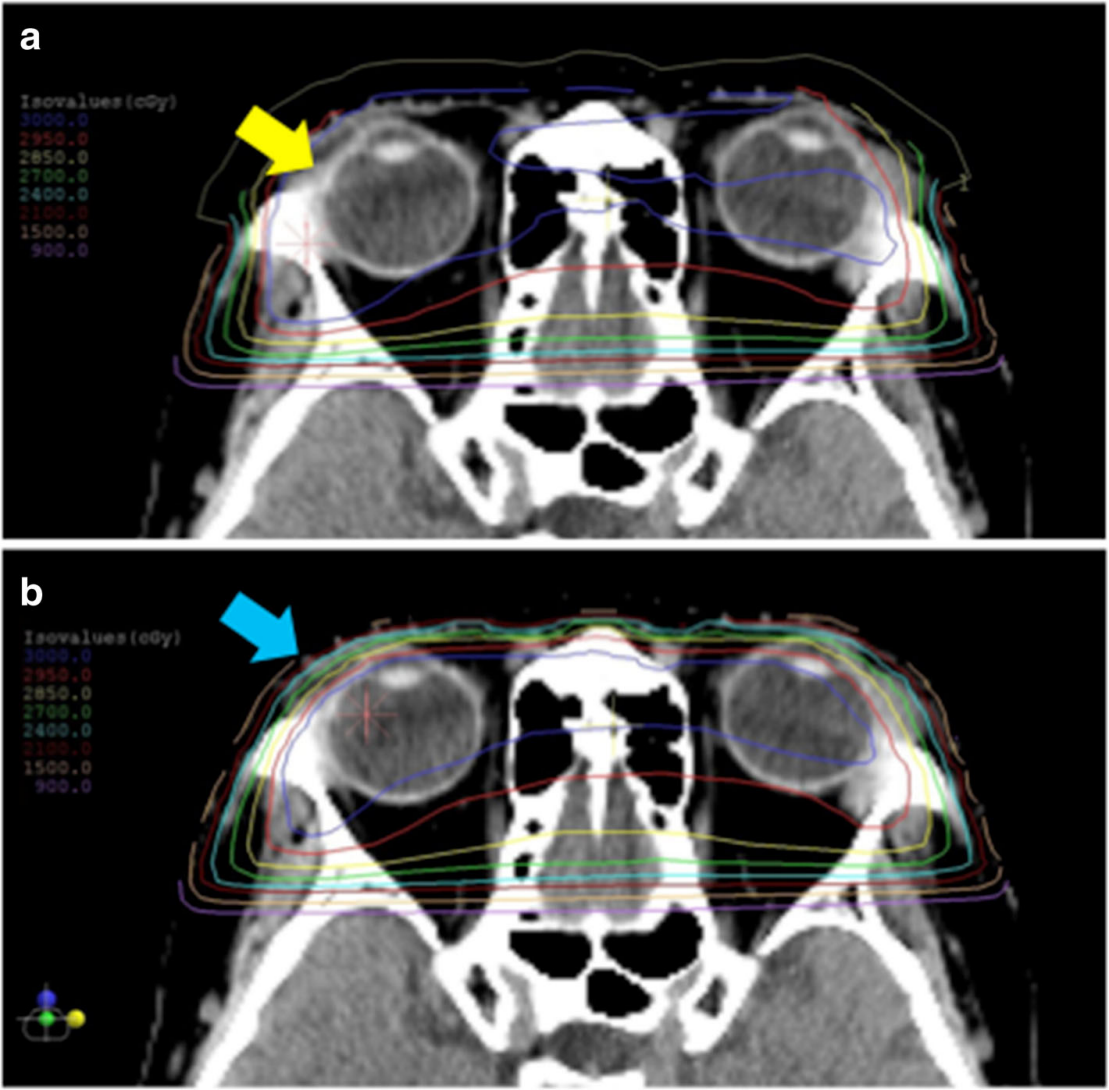
The comparison of dose distributions obtained with 6-MV photon beam with 5 mm bolus (**a**), and without bolus (**b**) for a bilateral case. Blue, red, yellow bright green, bright blue, dark red, orange and violet lines represent 3000 cGy, 2950 cGy, 2850 cGy, 2700 cGy,2400 cGy, 2100 cGy, 1500 cGy and 900 cGy isodose line, respectively. The surface of the right eye is covered more than 3000 cGy in A (yellow arrow) but not covered in B (blue arrow)

In this study, the prescribed doses were 30 to 30.6Gy, and all cases were treated without lens shielding. However, the dose distribution within the treatment field is not homogeneous; therefore, the dose to the lenses would vary in each patient. The dose to the surface of a patient is relatively low in single photon beam treatment or even electron beam treatment. To compensate for the surface dose, a bolus, which is water or near-water equivalent material, was placed on the surface of the patient. A bolus is used for radiotherapy for orbital malignant lymphoma when it needs to increase the dose to the surface of patients in both electron and photon beam treatments [25, 26]. In addition to electron beam and single photon beam treatments, we used a bolus for a bilateral case with opposed lateral fields. Nine eyes were treated with bolus and twelve were without bolus in this series. Figure 2 is the simulation planning for a bilateral case to compare the dose distribution with and without bolus. Example 1 is the dose distribution with 5 mm bolus and example 2 is without bolus (Fig. 2). In example1, the bolus technique increased the surface dose compared to example 2. When the bolus is used for ocular adnexal MALT lymphoma, the dose to the surface; including the lens reaches almost 100% of the prescribed dose. It is considered that the dose to the optic lens was higher in cases with than those without a bolus. Seven eyes treated with the bolus eventually developed cataract that required surgery. In contrast, 2 eyes in the non-cataract group underwent a bolus technique (*P* < 0.05). Although it is useful to control MALT lymphomas, the bolus technique may be a risk factor for radiation cataract.

The general assumption is that cataract surgery is an event with relatively low morbidity and therefore the radiation cataract is often underestimated as a mild complication of radiation therapy. However, a cataract surgery in a relatively younger age can cause inconvenience to the patient such as the early loss of accommodation ability, or unsymmetrical vision if the radiation cataract was induced unilatelally in patients with unilateral MALT lymphoma. Moreover, in this study, there were 11 cases of cataract surgery and 3 of those involved complications related to or subsequent to the extraction. There may be higher prevalence of complications of cataract surgery in radiation cataract, compared to senile cataract. Although it is difficult to assess that prevalence in this study, it may be important to evaluate the outcome of surgery in radiation-induced cataract in future studies.

The most crucial limitation of this study was the relatively small number of cases examined. Also, we could not consider the effect of lens shielding as the technique was not applied at radiotherapy for any of the patients. Further analysis with a larger cohort is required. Also, in many previous reports about radiation cataract, LOC III (Lens Opacities Classification System III) [27] or Merriam-Focht scoring system [28] are often used as a grading system. Also, others recommend Scheimpflug imaging system [24]. However, since this study is a retrospective study, and we usually do not use retroilumination or Scheimpflug imaging system in clinical practice, it was difficult to apply the system. Finally, in the previous studies describing detailed profile of radiation cataract in ocular adnexal MALT lymphoma [7, 8], the median follow-up duration is 66 months and 9 years, respectively. Comparing to them, we consider our median follow-up duration of 58.7 months is rather short. A study with longer follow-up period is needed to understand the prevalence of the radiation cataractgenesis in ocular adnexal MALT lymphoma.

## Conclusions

Our results showed that more than half of the patients after radiotherapy against ocular adnexal MALT lymphoma required cataract surgery. Patients with ocular adnexal MALT lymphoma who underwent surgery for radiation cataract were relatively young, and surgery was required about 3 years after radiotherapy. This indicates that relatively long-term observation may be mandatory for patients after radiotherapy. A female sex and the use of a bolus technique in radiation therapy may be risk factors for radiation cataract.

## Abbreviation

MALT: Mucosa-associated lymphoid tissue
RT: Radiation therapy
